# Covered Endovascular Reconstruction of Aortic Bifurcation Facilitated by Intravascular Lithotripsy With Shockwave Balloon: A Case Report

**DOI:** 10.7759/cureus.66874

**Published:** 2024-08-14

**Authors:** Leonardo Misuraca, Angela Buonpane, Giancarlo Trimarchi, Andrea Picchi, Federico Filippi, Antonio Biancofiore, Ugo Limbruno, Antonio Rizza

**Affiliations:** 1 Department of Interventional Cardiology, Misericordia Hospital, Azienda USL (Unità Sanitaria Locale) Toscana Sud Est, Grosseto, ITA; 2 Department of Cardiology, Foundation University Polyclinic Agostino Gemelli, Istituto di Ricovero e Cura a Carattere Scientifico (IRCCS), Rome, ITA; 3 Department of Clinical and Experimental Medicine, University of Messina, Messina, ITA; 4 Department of Cardiology, Heart Hospital, Fondazione Toscana Gabriele Monasterio, Massa, ITA

**Keywords:** shockwave intravascular lithotripsy, cerab, intravascular ultrasound, covered endovascular reconstruction of aortic bifurcation, peripheral vascular disease

## Abstract

Aortoiliac occlusive disease (AIOD) is a specific form of peripheral artery disease (PAD) that affects the infrarenal aorta and iliac arteries. Patients with PAD commonly suffer from intermittent claudication (IC), a condition characterized by cramping pain during or after exercise that is relieved by rest. The first-line therapy for IC involves medical management, foot care, and structured exercise programs while revascularization therapy, which can be endovascular, surgical, or a combination of both, is generally reserved for patients with claudication who do not respond adequately to initial therapies. We present the clinical case of a 58-year-old female with hypertension, dyslipidemia, and a smoking habit who was referred to our hospital (Misericordia Hospital, Grosseto, Italy) due to bilateral IC of the buttocks and thighs. Computed tomography (CT) angiography revealed a 90% tight stenosis of the infrarenal abdominal aorta just above the iliac bifurcation with diffuse calcifications. After a careful evaluation of the patient's condition and anatomical characteristics of the atherosclerotic disease, the vascular team decided to perform covered endovascular reconstruction of aortic bifurcation (CERAB) with previous intravascular lithotripsy (IVL) with shockwave balloon using intravascular ultrasound (IVUS) as guidance, because of severe aortic luminal calcifications. We performed successful CERAB, and the patient was discharged in good clinical condition on the fifth day of hospitalization with an indication to follow optimal medical therapy. At the one-month clinical follow-up, the patient reported the disappearance of claudication with marked improvement in quality of life. This first described case of IVUS-guided IVL-facilitated CERAB demonstrates the efficacy and safety of IVL in calcific aortic disease and shows the usefulness of IVUS as guidance in peripheral calcium debulking procedures.

## Introduction

Aortoiliac occlusive disease (AIOD) is a specific form of peripheral artery disease (PAD) that affects the infrarenal aorta and iliac arteries. The clinical presentation of patients with PAD can be categorized into four clinical subsets: asymptomatic PAD, chronic symptomatic PAD, chronic limb-threatening ischemia (CLTI), and acute limb ischemia (ALI) [1]. Patients with asymptomatic PAD may have just functional impairment, while chronic symptomatic PAD is typically characterized by intermittent claudication (IC), a condition characterized by cramping pain during or after exercise that is relieved by rest. CLTI and ALI are severe clinical subsets of PAD, respectively characterized by rest pain, nonhealing ulcers, or gangrene with symptoms present for >2 weeks and acute clinical symptoms (<2 weeks) with pain, pallor, pulselessness, and paresthesia [1].

In the subset of chronic symptomatic PAD, the first-line therapy involves medical management, foot care, and structured exercise programs. Revascularization therapy, which can be endovascular, surgical, or a combination of both, is generally reserved for patients with claudication who do not respond adequately to initial therapies and the choice of revascularization strategy depends on patient clinical status, anatomical lesion characteristics, operator's experience and the technologies available [1,2]. According to current guidelines, endovascular revascularization is the first choice to improve walking performance in patients with IC suffering from AIOD and femoro-popliteal disease, while surgical revascularization is a reasonable alternative for patients with low surgical risk if technical factors suggest it may offer advantages over endovascular methods [1]. 

The covered endovascular reconstruction of the aortic bifurcation (CERAB) technique, introduced in 2009, revolutionized aortic intervention by offering a sophisticated method for addressing complex aortic bifurcation pathologies. This innovative approach involves the strategic placement of bare or covered stents in the infrarenal aorta, followed by proximal ballooning to create a cone-shaped stent. Subsequently, two covered stents are precisely deployed in the distal conic segment at the aortic bifurcation level, with simultaneous inflation. This procedure not only ensures optimal anatomical reconstruction but also facilitates more physiological blood flow dynamics, surpassing conventional techniques like the kissing stent (KS) approach [3]. In this clinical case of infrarenal abdominal aorta calcified disease, we performed effective CERAB technique with previous intravascular lithotripsy (IVL) with shockwave balloon using intravascular ultrasound (IVUS) as guidance. 

## Case presentation

**Figure 1:**
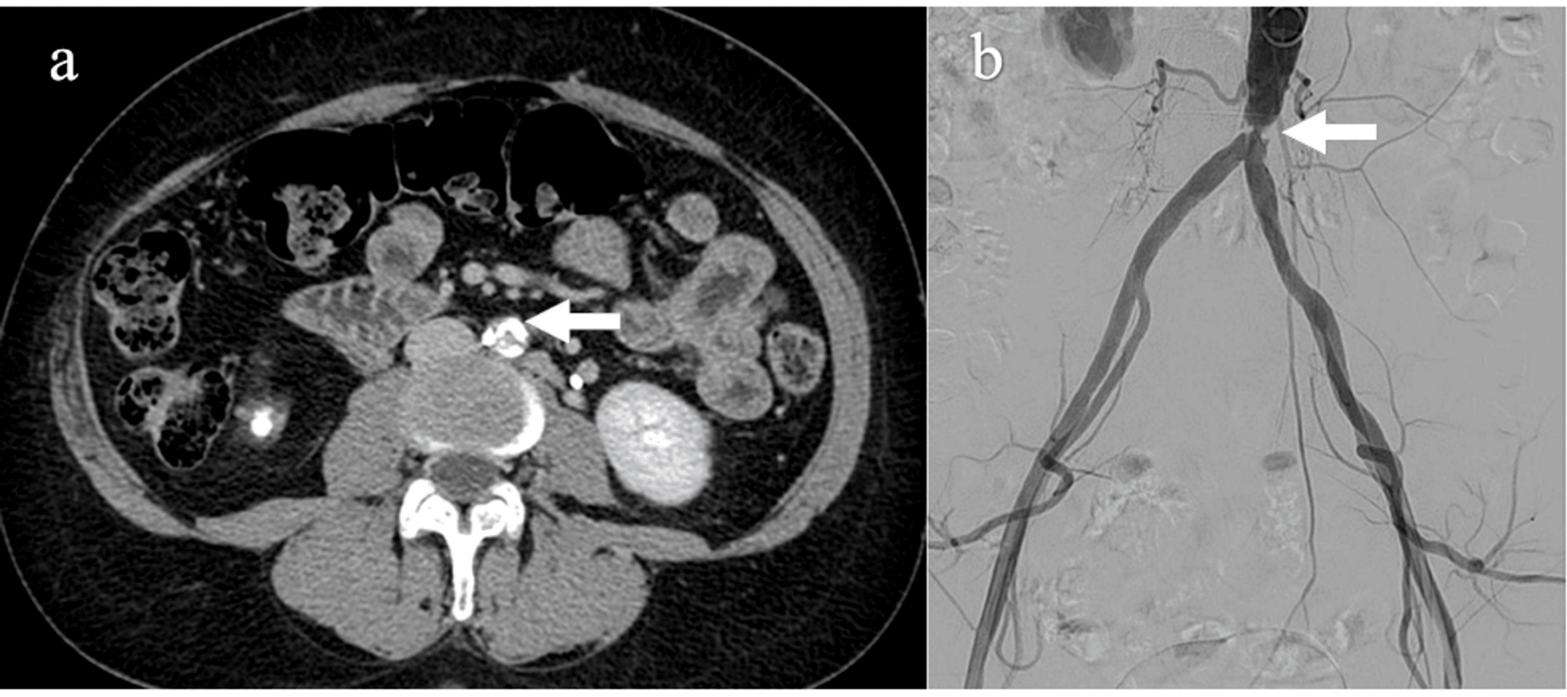
(a) Angio-CT scan, stenosis of the infrarenal abdominal aorta with severe calcifications (arrow); (b) angiography, stenosis of the distal abdominal aorta (arrow).

A 58-year-old female with hypertension, dyslipidemia, and a smoking habit was referred to our hospital (Misericordia Hospital, Grosseto, Italy) due to bilateral intermittent claudication of the buttock and thighs she had been suffering for two months, with the onset of pain at distances less than 200 meters (Fontaine IIb and Rutherford 3 scales) [4,5] and relief at rest. Her previous medical history was significant for an acute myocardial infarction with no obstructive coronary artery disease six years before and right renal nephrectomy because of massive nephrolithiasis. The patient's home therapy consisted of acetylsalicylic acid 100 mg daily, rosuvastatin 20 mg daily, ramipril 5 mg daily, ranolazine 500 mg daily, pantoprazole 40 mg daily, cilostazol 100 mg twice daily and home-based exercise therapy. Physical examination revealed decreased femoral pulses with an ankle-brachial index (ABI) of 0.7, without gangrene or lower limb ulceration. Computed tomography (CT) angiography revealed a 90% tight stenosis of the infrarenal abdominal aorta just above the iliac bifurcation with diffuse, semi-concentric calcifications (Figure 1a).

Several treatment options were considered by the vascular team: surgical repair with bypass, traditional endovascular treatment with the KS technique, and CERAB. The choice of the best treatment was based on the patient's clinical condition, CT angiography anatomical findings, operators’ expertise, and patient's preference. In particular, the vascular team considered the anatomical findings, which according to the Trans-Atlantic Inter-Society Consensus (TASC) II classification fall into type B, a short stenosis (less than 3 cm) of the infrarenal abdominal aorta, for which TASC II, European Society of Vascular Surgery (ESVS) and American College of Cardiology/American Heart Association guidelines recommend endovascular treatment [1,2,6]. Finally, in agreement with the patient, it was decided to perform the CERAB procedure. A 4 Fr sheath was percutaneously inserted in the left brachial artery inserted in the left brachial artery and a pigtail catheter was advanced into the abdominal aorta. Digital subtraction angiography confirmed a severe stenosis of the distal abdominal aorta (Figure 1b).

**Figure 2:**
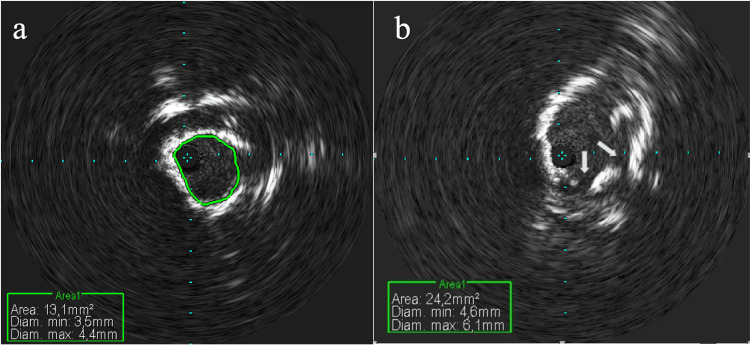
(a) Intravascular ultrasound, semi-concentric calcifications at the level of the stenosis; (b) after intravascular lithotripsy, intravascular ultrasound demonstrated calcium debulking (arrows) with an enlarged lumen area.

**Figure 3:**
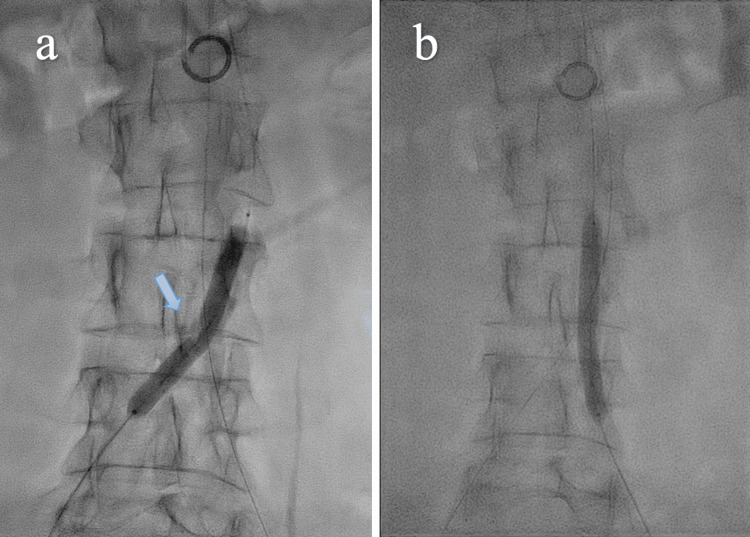
(a) Intravascular lithotripsy (IVL) balloon inflated on the right side, waist of the balloon (arrow); (b) intravascular lithotripsy balloon inflated on the left side.

The right common femoral artery was percutaneously cannulated with a 9 Fr sheath and the left common femoral artery with a 7 Fr sheath. Perclose™ ProStyle™ Suture-Mediated Closure and Repair (SMCR) system (Abbott, Abbott Park, IL, USA) was positioned in both artery accesses. A 0.014 supportive wire was advanced retrogradely in the ascending aorta through the right femoral sheath. IVUS pullback was performed with a Volcano Visions PV .035 Digital IVUS catheter (Philips, San Diego, CA, USA) showing widespread calcifications of the infrarenal abdominal aorta with circumferential distribution. After IVUS evaluation (Figure 2a) and advancement of a second 0.014 wire through the left femoral introducer, we performed IVL with an M5 + 7.0 x 60 mm shockwave balloon (4 atm, 50-60 Hz, 30 pulses, four cycles for a total of 120 pulses) (Figure 3a), advanced via the right side. After four cycles of IVL, the balloon was removed from the right side and readvanced via the left side, delivering four additional cycles (4 atm, 50-60 Hz 30 pulses, four cycles for a total of 120 pulses) (Figure 3b). The IVUS pullback revealed effective calcium debulking with an enlarged lumen area (Figure 2b).

**Figure 4:**
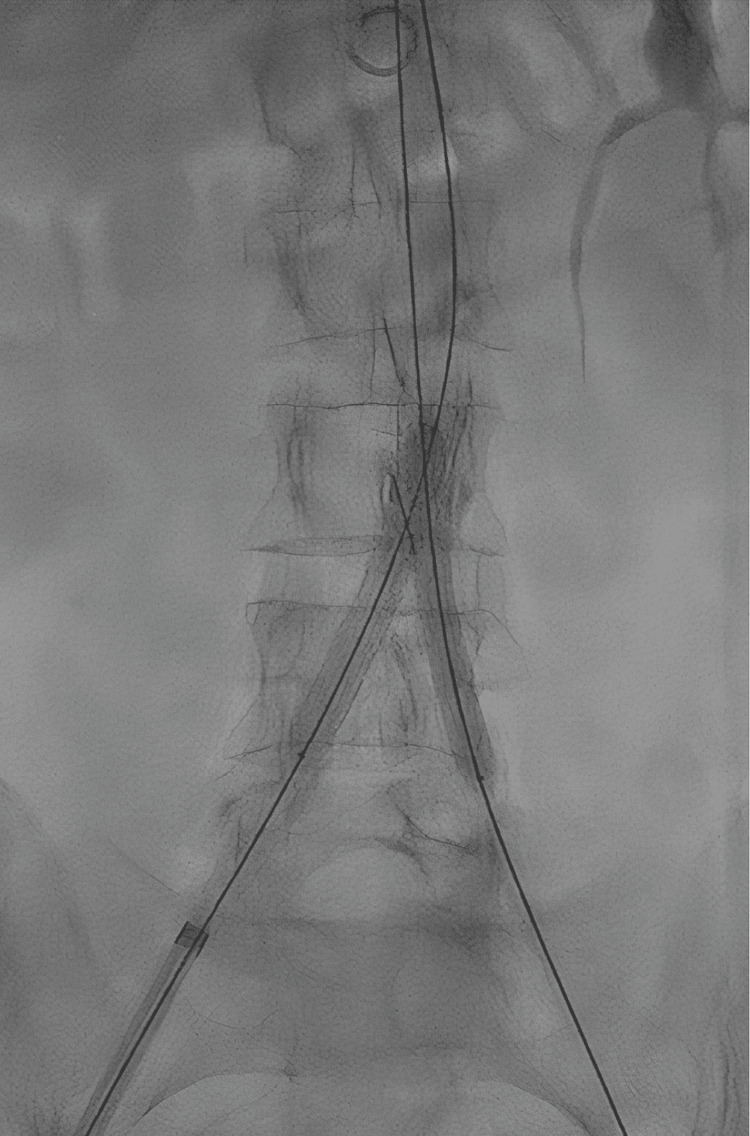
Simultaneous implantation of two covered stents.

No signs of aortic wall dissection or rupture were detected at control angiography. At this point, the 9 Fr right femoral sheath was exchanged with a 12 Fr long introducer, whose tip was advanced beyond the lesion. A 14 mm x 29 mm covered, balloon expandable, stent was implanted about 20 mm above the aortic bifurcation (BeGraft aortic stent, Bentley InnoMed, Hechingen, Germany). Two covered stents (8.0 x 59 mm on the right, 7.0 x 59 mm on the left side) were simultaneously implanted from the distal half of the aortic stent down to the common iliac arteries (BeGraft peripheral, Bentley, Hechingen, Germany) (Figure 4).

**Figure 5:**
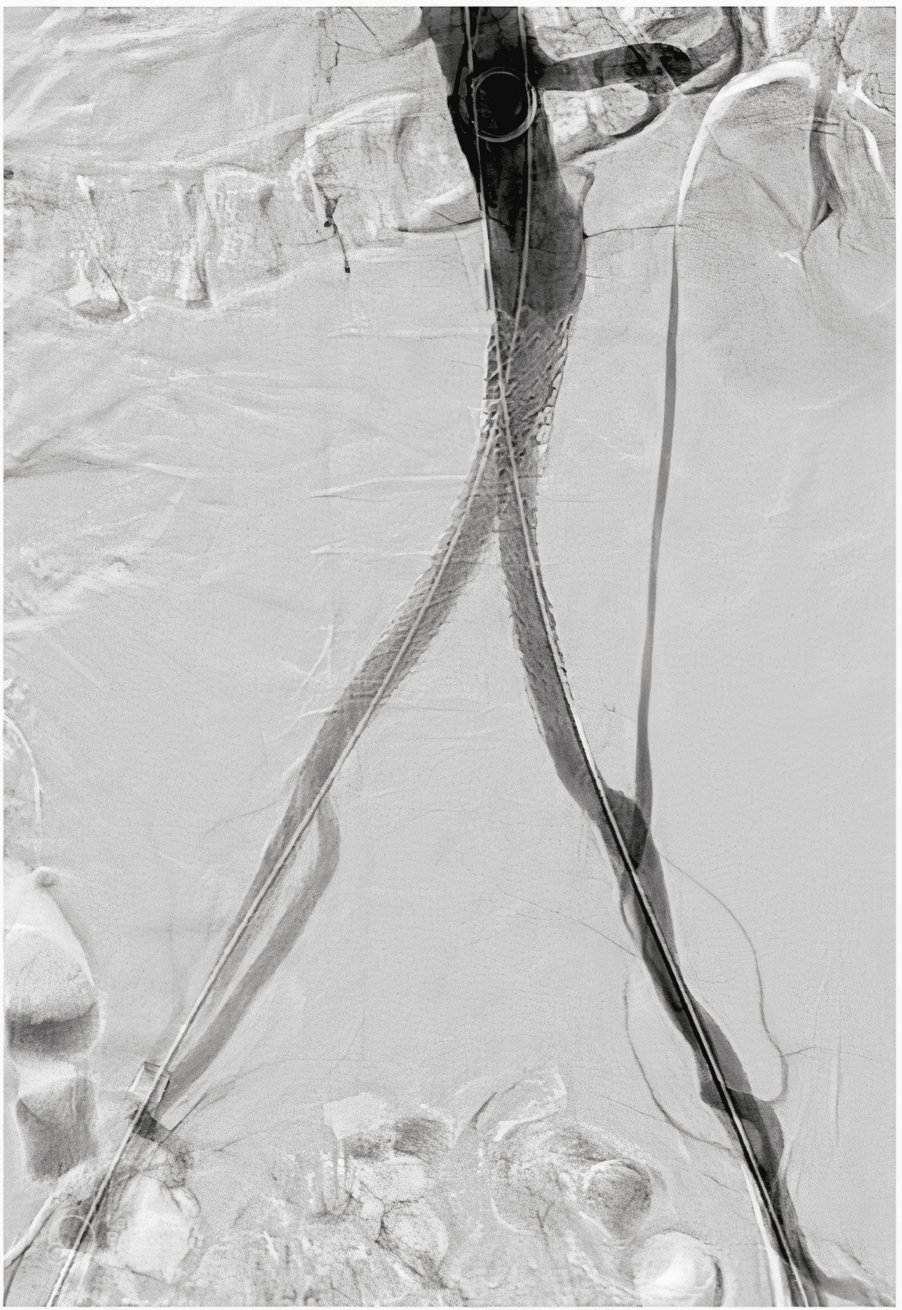
Angiography, final result.

Postdilation with kissing balloon inflation was performed. Final angiography showed excellent results (Figure 5), with appropriate expansion and apposition of the covered stent to the aortic wall. Final hemostasis of the right and left femoral artery was achieved using Perclose™ ProStyle™ SMCR.

The post-procedure course was free of complications and the patient was discharged in good clinical condition on the fifth day of hospitalization with an indication to follow dual antiplatelet therapy (DAPT) with Cardioaspirin 100 mg daily and clopidogrel 75 mg daily for three months and with ezetimibe 10 mg daily in addition to rosuvastatin 20 mg. The patient was given careful recommendations about lifestyle, control of cardiovascular risk factors, and cessation of the smoking habit. At one-month clinical follow-up, the laboratory examinations were in the normal range with low-density lipoprotein (LDL) cholesterol levels at target (<55 mg/dl), physical examination revealed an ABI of 1 with normal femoral pulses, and the patient reported disappearance of claudication with marked improvement in quality of life and cessation of the smoking habit.

## Discussion

CERAB has recently gained popularity as an endovascular treatment AIOD. According to current guidelines, in patients with intermittent claudication, endovascular treatment is now preferred over open surgery, which is reserved for more complex anatomical scenarios and for younger patients with low surgical risk and a longer life expectancy [1,2]. In this scenario, various revascularization techniques have been developed, including the use of balloon-expandable stents, self-expanding stents, covered stents, and even balloon angioplasty alone. For many years, the KS technique has been employed in the treatment of AIOD. This technique involves placing bilateral iliac stents parallel to each other in the aorta and inflating them simultaneously, with the proximal ends of the stents extending into the distal aorta [3].

The CERAB technique consists of displacing the aortic bifurcation cranially, by implanting one balloon-expandable covered aortic stent about 15-20 mm above the bifurcation and, subsequently, two iliac-covered stents from the distal portion of the aortic stent down to the common iliac arteries [7]. It ensures optimal anatomical reconstruction but also facilitates more physiological blood flow dynamics, overcoming conventional techniques like the KS approach [3].

A recent meta-analysis involving 681 patients, with lesions located from the iliac to the below-the-knee district demonstrated good results (59.3 % of diameter stenosis reduction) and excellent safety (major dissections in 1.25% of cases) of IVL in peripheral artery disease [8]. IVL employs acoustic shockwaves administered via a semi-compliant balloon to fracture and disintegrate intimal and medial calcification. This technique allows effective calcium debulking with minimal inflation pressure, thereby mitigating the likelihood of complications associated with barotrauma, and appears to be even more effective in treating lesions with characteristics that typically result in poorer clinical outcomes following endovascular interventions, such as thicker and more circumferential calcium [9]. Using a single lithotripsy balloon, undersized compared to the aortic diameter, but well-sized according to the stenosis diameter, inflated alternatively via the right and the left side, we avoided the use of large balloons/kissing balloons furthermore reducing the risk of aortic wall injury during calcium debulking. Several studies have highlighted the successful application of IVL during transcatheter aortic valve replacement (TAVR) [10,11], endovascular aneurysm repair (EVAR), thoracic endovascular aneurysm repair (TEVAR) and CERAB [12-14] in the context of narrowed and severely calcified aortic and iliac arteries, which represents a significant challenge for the use of large-bore devices due to reduced vessel compliance and increased rigidity.

In this case, we performed IVUS-guided IVL. IVUS is a diagnostic tool with a pivotal role in both coronary and peripheral interventions offering a more comprehensive assessment of atherosclerotic disease compared to angiography alone, which, although the dominant imaging modality in revascularization, has inherent limitations as it provides only two-dimensional images of three-dimensional structures [15]. It enables precise measurement of lumen diameter, lumen cross-sectional area, and the size of the reference vessel, providing essential guidance for treatment decisions. Additionally, IVUS allows the characterization of plaque morphology and is particularly valuable in identifying and grading calcification, serving as a guide in the treatment of calcified lesions and in the planning of calcium debulking strategies [15].

## Conclusions

Covered endovascular reconstruction of aortic bifurcation (CERAB) has recently gained popularity as an endovascular treatment for aortoiliac occlusive disease. This technique is performed by placing a bare or covered stent in the infrarenal aorta, proximally ballooned, and deploying two covered stents in the distal segment at the aortic bifurcation level with simultaneous inflation. When CT reveals severe calcification of the infrarenal abdominal aorta, techniques such as intravascular lithotripsy (IVL) may be employed to facilitate stent deployment. IVL with shockwave balloon can modify vessel compliance with low inflation pressure reducing risks of barotrauma-related complications. Intravascular ultrasound (IVUS) plays a pivotal role as a diagnostic tool to identify and grade calcification and serves as guidance in planning calcium debulking strategies and treating calcified lesions. In this clinical case, the use of IVUS allowed us to identify calcium burden and distribution, choose the correct calcium debulking strategy, and verify its effectiveness. To the best of our knowledge, this is the first described case of IVL-facilitated CERAB.
